# Low Level of Sequence Diversity at Merozoite Surface Protein-1 Locus of *Plasmodium ovale curtisi* and *P. ovale wallikeri* from Thai Isolates

**DOI:** 10.1371/journal.pone.0058962

**Published:** 2013-03-11

**Authors:** Chaturong Putaporntip, Austin L. Hughes, Somchai Jongwutiwes

**Affiliations:** 1 Molecular Biology of Malaria and Opportunistic Parasites Research Unit, Department of Parasitology, Faculty of Medicine, Chulalongkorn University, Bangkok, Thailand; 2 Department of Biological Sciences, University of South Carolina, Columbia, South Carolina, United States of America; Universidade Federal de Minas Gerais, Brazil

## Abstract

**Background:**

The merozoite surface protein-1 (MSP-1) is a candidate target for the development of blood stage vaccines against malaria. Polymorphism in MSP-1 can be useful as a genetic marker for strain differentiation in malarial parasites. Although sequence diversity in the MSP-1 locus has been extensively analyzed in field isolates of *Plasmodium falciparum* and *P. vivax*, the extent of variation in its homologues in *P. ovale curtisi* and *P. ovale wallikeri*, remains unknown.

**Methodology/Principal Findings:**

Analysis of the mitochondrial cytochrome *b* sequences of 10 *P. ovale* isolates from symptomatic malaria patients from diverse endemic areas of Thailand revealed co-existence of *P. ovale curtisi* (n = 5) and *P. ovale wallikeri* (n = 5). Direct sequencing of the PCR-amplified products encompassing the entire coding region of *MSP-1* of *P. ovale curtisi* (*PocMSP-1*) and *P. ovale wallikeri* (*PowMSP-1*) has identified 3 imperfect repeated segments in the former and one in the latter. Most amino acid differences between these proteins were located in the interspecies variable domains of malarial MSP-1. Synonymous nucleotide diversity (π_S_) exceeded nonsynonymous nucleotide diversity (π_N_) for both *PocMSP-1* and *PowMSP-1*, albeit at a non-significant level. However, when MSP-1 of both these species was considered together, π_S_ was significantly greater than π_N_ (p<0.0001), suggesting that purifying selection has shaped diversity at this locus prior to speciation. Phylogenetic analysis based on conserved domains has placed *PocMSP-1* and *PowMSP-1* in a distinct bifurcating branch that probably diverged from each other around 4.5 million years ago.

**Conclusion/Significance:**

The MSP-1 sequences support that *P. ovale curtisi* and *P. ovale wallikeri* are distinct species. Both species are sympatric in Thailand. The low level of sequence diversity in *PocMSP-1* and *PowMSP-1* among Thai isolates could stem from persistent low prevalence of these species, limiting the chance of outcrossing at this locus.

## Introduction

About half of the world's population resides in areas at risk of contracting malaria, one of the leading causes of morbidity and mortality, accounting for more than 200 million cases and more than 600,000 deaths per annum [1]. Six species in the genus *Plasmodium* are known to cause human malaria under natural transmission [2]–[4]. However, only malaria caused by *Plasmodium falciparum* and *Plasmodium vivax* have been extensively studied, whereas relatively little is known about the less prevalent malaria parasites. Although *Plasmodium ovale* has been proposed as a valid human malaria species by Stephens in 1922 upon examination of the blood sample from a patient acquiring infection from East Africa [5], it was not until almost a century later that this malaria has been further subdivided into 2 distinct species, i.e. *P. ovale curtisi* (or classic type) and *P. ovale wallikeri* (or variant type) based on molecular analysis [4], [6], [7].

*P. ovale* has a wide geographic distribution across tropical countries, especially Africa, Asia and some Western Pacific islands [8]. In several malaria endemic areas, *P. ovale* has been found to be sympatric with the major malaria species, *P. falciparum* and *P. vivax*. The low parasite densities of *P. ovale* in infected individuals and its morphological resemblance to *P. vivax* have hampered efficient microscopy detection, especially when they co-exist with other malaria species in circulation. Therefore, the actual prevalence of this malaria species can be underestimated [8], [9]. Based on limited epidemiological data, it has been estimated that at least 15 million cases occur annually in Sub-Saharan African countries [4]. Because *P. ovale* possesses a hypnozoite stage in liver cells similar to that found in *P. vivax*, a relapsing course of infection can ensue.

To date, relatively few molecular markers are available to document the extent of genetic variation and strain differentiation of the sibling species of *P. ovale*. One of the polymorphic genetic loci that has been well characterized in *P. falciparum* and *P. vivax* is the gene encoding the merozoite surface protein-1 (MSP-1) [10], [11]. The precursor of *P. falciparum* merozoite surface protein-1 (PfMSP-1) is synthesized during schizogony and undergoes primary processing that generates polypeptides of 83, 30, 38 and 42 kDa [12]. The 42-kDa fragment at the C-terminus is further proteolytically cleaved into 33 and 19 kDa fragments by the time of erythrocyte entry. The 19 kDa fragment, containing two epidermal growth factor (EGF)-like motifs, remains attached to the surface of newly invasive merozoite through the ring stage whereas other processed fragments are shed in circulation [12]. Besides being one of the prime asexual erythrocytic vaccine candidates, MSP-1 exhibits extensive sequence divergence within and between different malaria species [13]. Therefore, analysis of this genetic locus will be useful for detailed characterization of the two sibling species of *P. ovale*.

Recently, two nucleotide sequences of the gene encoding the merozoite surface protein-1 of *P. ovale curtisi* (*PocMSP-1*) from Cameroon patients were determined [14]. However, the MSP-1 sequence of *P. ovale wallikeri* (*PowMSP-1*) remains unknown. Herein, we have determined the extent of sequence variation in the *MSP-1* locus of isolates derived from symptomatic malaria patients in Thailand whose blood samples contained *P. ovale* based on polymerase chain reaction (PCR)-based detection targeting the small subunit ribosomal RNA gene. Sequence analysis has led to identification of the MSP-1 sequence of *P. ovale wallikeri* in Thai patients.

## Materials and Methods

### Human Ethics Statement

The protocol was reviewed and approved by the Institutional Review Board on Human Research of Faculty of Medicine, Chulalongkorn University (IRB259/54). Written informed consent was obtained from participants or from parents/legally guardians enrolled using an approved consent form.

### P. ovale isolates

Blood samples with single infection of *P. ovale* were obtained from 10 symptomatic malaria patients who acquired the infections from diverse endemic areas of Thailand. Background data of each isolate was shown in Table 1. DNA from each isolate was prepared by using Qiagen DNA mini kit (Qiagen, Hilden, Germany) following the protocol provided by the manufacturer and stored at −40 °C until use. Diagnosis of *P. ovale* was performed by both microscopy and nested PCR targeting the small subunit ribosomal RNA genes (*SSU rRNA*) of 5 human malaria species as previously described [15], [16]. The diagnostic primers for *P. ovale* could amplify the *SSU rRNA* genes of both classic and variant types.

**Table 1:** Demographic and parasitologic profiles of patients infected with *Plasmodium ovale*.

Isolate	Age (Year)	Sex	Year infected	Place acquiring infection	Parasite density (/µl)	*SSU rRNA*-PCR	*mtCYTB* sequence*
PO-1	27	Male	1993	Kanchanaburi Province	2,400	*P. ovale*	*P. ovale wallikeri*
PO-2	28	Male	1994	Kanchanaburi Province	3,200	*P. ovale*	*P. ovale wallikeri*
PO-3	33	Male	1994	Kanchanaburi Province	2,520	*P. ovale*	*P. ovale wallikeri*
PO-4	19	Female	1995	Tak Province	1,000	*P. ovale*	*P. ovale curtisi*
PO-5	14	Female	2006	Tak Province	2,560	*P. ovale*	*P. ovale wallikeri*
PO-6	35	Female	2007	Tak Province	600	*P. ovale*	*P. ovale wallikeri*
PO-7	12	Male	2008	Kanchanaburi Province	1,280	*P. ovale*	*P. ovale curtisi*
PO-8	30	Male	2010	Tak Province	3,520	*P. ovale*	*P. ovale curtisi*
PO-9	23	Male	2010	Tak Province	2,560	*P. ovale*	*P. ovale curtisi*
PO-10	34	Male	2010	Yala Province	1,760	*P. ovale*	*P. ovale curtisi*

* Nucleotide differences between the *mtCYTB* fragment occurring at respective positions 175, 187, 205, 316 and 334 were G, C, T, C and C for *P. ovale curtisi* and T, T, A, T and T for *P. ovale wallikeri*.

### PCR amplification and sequencing of the mitochondrial cytochrome b gene

A fragment of the mitochondrial cytochrome *b* (mtCYTB) gene of *Plasmodium ovale* was amplified by PCR using primers PoCytbF (5′-CTTACATTTACATGGTAGAC-3) and PoCytbR (5′-GCCATTTTGAATTGTATAATAG-3′) in a total volume of 25 µL containing 1 µL of template DNA, 2.5 mM each deoxynucleoside triphosphate, 2.5 µL of 10× PCR buffer, 0.3 µM of each primer and 0.5 unit of ExTaq DNA polymerase (Takara, Seta, Japan). The thermal cycling profile included a preamplification denaturation at 94°C, 1 min; 35 cycles of denaturation at 94°C, 40 s, annealing at 53°C, 30 s and extension at 72°C, 30 s; and post amplification extension at 72°C, 5 min. All amplification reactions were done in an Applied Biosystem GeneAmp® PCR System 9700 thermocycler (PE Biosystems, Foster City, CA). PCR products were analyzed by 1% agarose gel electrophoresis. DNA sequencing was performed directly from the purified PCR product using Qiagen PCR purification kit (Qiagen, Hilden, Germany).

### PCR amplification and sequencing of the PoMSP-1 gene

The nucleotide sequence of *PoMSP-1* was amplified by nested PCR using PoMSP1F0 (5′-AATTCAAAAATGAAGGTGTTC-3′) and PoMSP1R0 (5′-CTTTTGTATTTACCCTCACTC-3′) as outer primers and PoMSP-1F1 (5′-AGGTGTTCGTATTTGCGCTC-3′) and PoMSP-1R1 (5′-CTCTCTCCTTTTAAAGTAAG-3) as inner primers. Two microlitres of the PCR products from primary PCR were used as template for secondary PCR in a total volume of 30 µl. Amplification conditions for primary and secondary PCRs were identical comprising 35 cycles of 96°C for 20 s, 62°C for 5 min with an initial pre-amplification denaturation at 94°C for 1 min and a final post-amplification extension at 72°C for 10 min. DNA sequences were obtained directly from the PCR-amplified products. Sequences have been deposited in the GenBank™ database under the accession numbers KC137340-KC137349.

### Data analysis

Alignment of the *PoMSP-1* nucleotide sequences was performed using the default option of the CLUSTAL_X program [17] and manually edited. Insertions/deletions (indels) in coding regions were determined from multiple alignments of amino acid sequences to maintain the reading frame. Sequences of the two Cameroon isolates (GenBank accession numbers FJ824670 and FJ824671) were included for comparison [14]. Tandem repeats were detected by scanning the sequence with a small window, determining the distance between exact matches and testing the statistical criteria as implemented in the Tandem Repeats Finder version 4.0 program [18]. Nucleotide diversity (π) was computed from the average number of pairwise sequence differences at synonymous sites (π_S_) and nonsynonymous sites (π_N_) in the sample sequences [19]. Standard errors of these parameters were estimated by the bootstrap method with 1,000 pseudoreplicates using the MEGA 5.05 program [20]. Significant differences between π_S_ and π_N_ by Z-tests were considered to provide evidence of selective pressure on tested regions. Nucleotide divergence between pairs of closely related malaria species was calculated from the number of base substitutions per site between sequences using the maximum composite likelihood model and its standard error was obtained by 1,000 bootstrap replicates. All sites with gaps were excluded from analysis. Sequences and their GenBank accession numbers included for analysis were the mtCYTB gene of *P. falciparum* (XM001348736), *P. rechenowi* (NC002235), *P. fieldi* (AB444133), *P. simiovale* (AY800109), *P. ovale curtisi* (GU723533) and *P. ovale wallikeri* (HQ712053); and the *SSU rRNA* (A-type) locus of these species (M19172, Z25819, AB287283, AB287287, JF894405 and JF894411, respectively). Phylogenetic tree was inferred from amino acid sequences by using the maximum likelihood method based on the Jones-Taylor-Thornton (JTT) model [21] as implemented in the MEGA 5.05 program [20]. Reliability of branching patterns was evaluated by bootstrapping using 1,000 iterations. The MSP-1 sequences of other malaria species and their accession numbers included for comparison were *P. falciparum* (X03371), *P. vivax* (AF435593), *P. malariae* (FJ824669), *P. knowlesi* (DQ220743), *P. fragile* (AB444067), *P. coatneyi* (AB266180), *P. inui* (AB444062), *P. hylobati* (AB266182), *P. cynomolgi* (AB444063), *P. fieldi* (AB444066), *P. simiovale* (AB266185), *P. gonderi* (AB444069), *P. chabaudi* (L22982), *P. berghei* (U43521), *P. yoelii* (XM721164), *P. gallinaceum* (AJ809338) and *P. reichenowi* (AJ786604). Estimation of divergence time between *PocMSP-1* and *PowMSP-1* was inferred from interspecies conserved domains by using the Bayesian Evolutionary Analysis by Sampling Trees (BEAST) package based on Markov Chain Monte Carlo (MCMC) algorithms [22]. The divergence time was calibrated with *PfMSP-1* and *PrMSP-1* assuming that *P. falciparum* and *P. reichenowi* have diverged along with their respective human and chimpanazee hosts since 6±0.5 million years ago (MYA). Analysis was performed by using uncorrelated lognormal relaxed clock, Yule Process for the tree prior, HKY site model with estimated base frequencies and a 4 category gamma site heterogeneity model. Simulations were run for 25,000,000 cycles and logged at every 1,000 cycles.

## Results

### Amplification and sequencing of mtCYTB of *P. ovale*

Amplification of the *mtCYTB* gene of *P. ovale* encompassing 357 bp fragment has generated single PCR products for all isolates examined. Among the ten isolates described here, sequence analysis identified 5 nucleotide substitutions that segregated perfectly into only two *mtCYTB* genotypes. The *mtCYTB* sequences of isolates PO-3, PO-4, PO-5, PO-6 and PO-8 contained G, C, T, C and C at positions 175, 187, 205, 316 and 334 (numbering from the first nucleotide in the forward amplification) consistent with *P. ovale curtisi* (GenBank accession number HQ712052) whereas the remaining isolates having T, T, A, T and T at these respective sites belonged to *P. ovale wallikeri* (accession number HQ712053) (Table 1).

### Amplification and sequencing of PoMSP-1

*PoMSP-1* was successfully amplified by PCR in all isolates, generating single PCR products of expected size (∼5 kb). Direct sequencing of these purified PCR fragments has shown no superimposed signal on electropherograms of *PoMSP-1*of these samples, suggesting no clonal mixtures in isolates examined. All 5 MSP-1 sequences of *P. ovale curtisi* contained 5,181 bp whereas 4 of 5 *P. ovale wallikeri* had 5,016 bp and the remaining isolate (PO-10) from southern Thailand possessed 5,043 bp. In total, 7 distinct *PoMSP-1* nucleotide sequences were identified among 10 Thai isolates. Perfectly identical sequences were observed in 3 isolates belonging to *P. ovale curtisi* (PO-4, PO-8 and PO-9) and 2 isolates identified as *P. ovale wallikeri* (PO-2 and PO-3).

### Comparison of MSP-1 from P. ovale curtisi and P. ovale wallikeri

**Figure 1:**
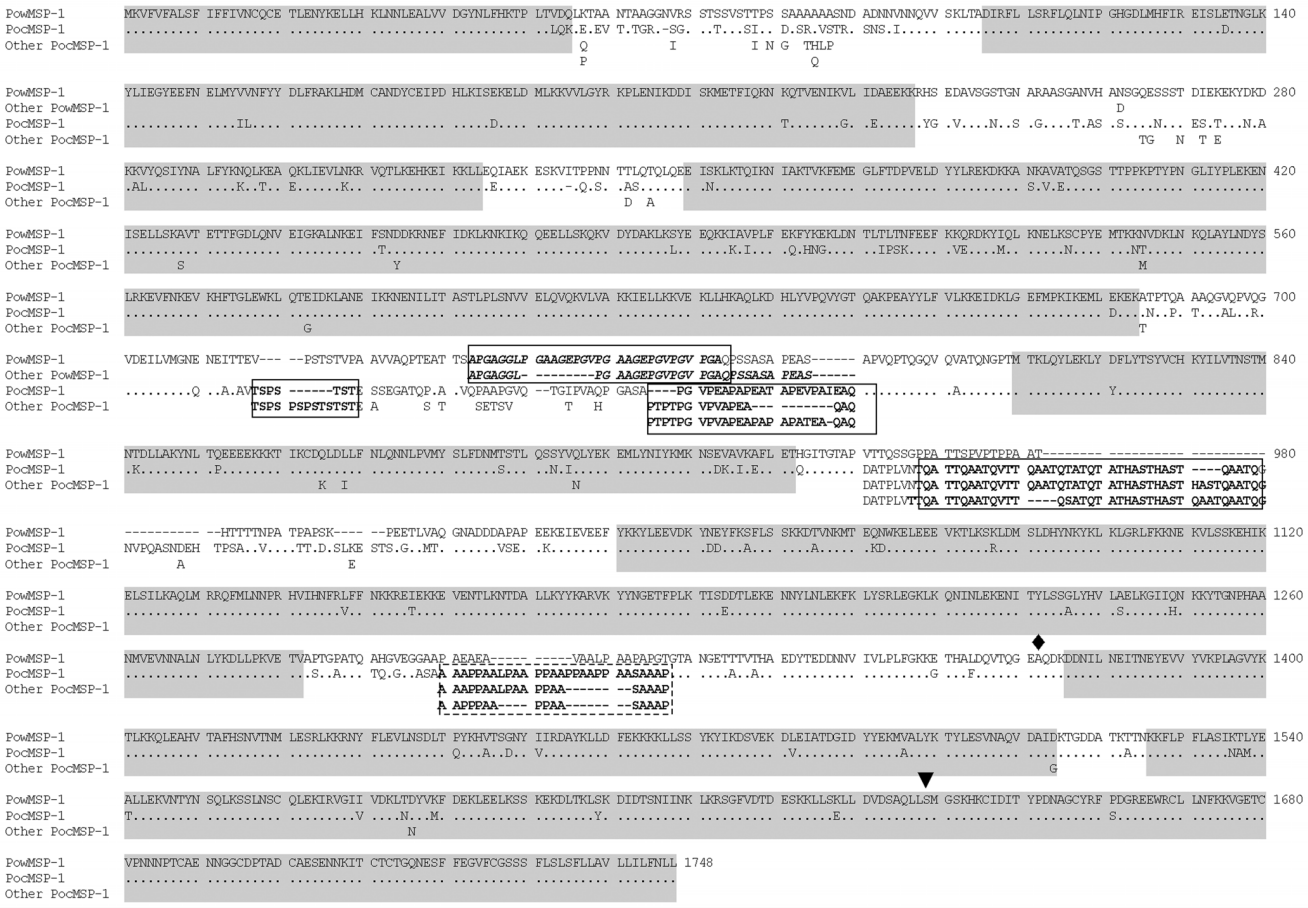
Alignment of amino acid sequences of PocMSP-1 and PowMSP-1 among 10 clinical isolates and two Cameroon strains (GenBank accession numbers HQ712052 and HQ712053). Dots and dashes represent residues identical to PowMSP-1 and deletions, respectively. ‘Other PocMSP-1’ and ‘Other PowMSP-1’ denotes possible substitutions in other isolates. Repeat regions are in bold, italicized and boxed. Interspecies conserved domains are shaded. Potential cleavage sites for generating 42 KDa- and 19 KDa-fragments are shown as diamond and arrow head above the alignment.

Sequence comparison of *MSP-1* derived from human, nonhuman primate, avian and murine malaria in previous studies have shown that malarial *MSP-1* could be partitioned into 15 domains comprising 7 variable domains flanked by interspecies conserved sequences [13], [14] (Figure 1). Alignment of the *PoMSP-1* sequences from Thai isolates has identified two distinct groups corresponding to each species as determined by the *mtCYTB* genotypes (Table 1). For *PoMSP-1* of *P. ovale curtisi* (*PocMSP-1*), 4 repeats-encoding regions were identified as shown in Figure 1. On the other hand, a 9-nucleotide repeat region having the consensus sequence AGGAGTACC was found in *PoMSP-1* of *P. ovale wallikeri* (*PowMSP-1*) that was conserved among isolates. In non-repeats regions, codon difference between *PocMSP-1* and *PowMSP-1* as well as insertion or deletion of amino acid residues were more pronounced outside interspecies conserved domains (95/311 codons, 30.55%) than those in interspecies conserved domains (87/1288 codons, 6.75%), consistent with the general pattern of interspecific sequence diversity of malarial MSP-1 locus. The potential cleavage sites generating 42 KDa and 19 KDa fragments were conserved among isolates. Although 38 nucleotide differences were detected in the 42 KDa-encoding fragments of both *PocMSP-1* and *PowMSP-1* resulting in 18 codon changes, these substitutions were conserved for each species. In the 19 KDa fragment, a single amino acid difference between *PocMSP-1* and *PowMSP-1* was identified, i.e. Ser1661Pro (position after Figure 1).

### Nucleotide diversity

Analysis of nucleotide substitution in *PocMSP-1* and *PowMSP-1* has shown that synonymous nucleotide diversity (π_S_) exceeded nonsynonymous nucleotide diversity (π_N_) (Table 2). However, these differences did not reach statistically significant levels. On the other hand, π_S_ significantly exceeded π_N_ (p<0.001) when both species were considered together, suggesting that purifying selection has shaped variation in the *PoMSP-1* locus preceding speciation, presumably from functional or structural constraint on the protein.

**Table 2:** Synonymous (π_S_) and nonsynonymous (π_N_) nucleotide diversity in non-repeat region of MSP-1.

	π_S_±S.E.	π_N_±S.E.
*P. ovale curtisi* (Thai and Cameroon isolates, n = 7)	0.0027±0.0013	0.0017±0.0005
*P. ovale curtisi* (Thai isolates, n = 5)	0.0013±0.0009	0.0001±0.0001
*P. ovale wallikeri* (Thai isolates, n = 5)	0.0005±0.0004	0.0000±0.0000
*P. ovale* (all)	0.0793±0.0080***	0.0160±0.0016

Z-tests of the hypothesis that π_S_ = π_N_: *** *p*<0.001.

When π_S_ and π_N_ were computed for all *P. ovale MSP-1*, including both *PocMSP-1* and *PowMSP-1*, the values were much greater than those computed for *PocMSP-1* and *PowMSP-1* separately (Table 2), as would be expected when combining data from two non-recombining species.

### Phylogenetic analysis and nucleotide divergence

**Figure 2:**
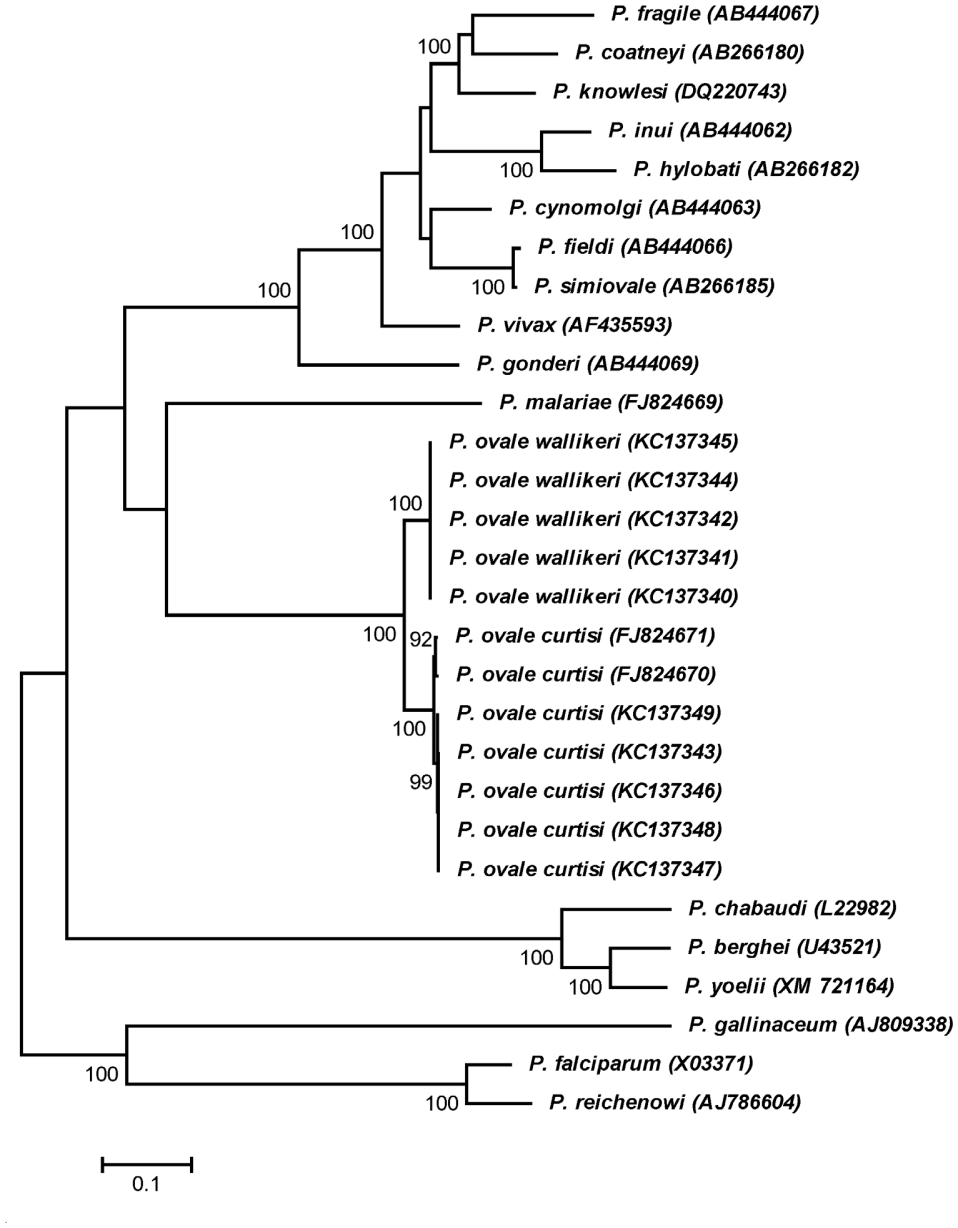
Neighbor-joining tree inferred from the MSP-1 intersepecies conserved sequences of human, nonhuman primate, avian and murine malaria. GenBank accession numbers are in paraentheses. Bootstrap values more than 50% based on 1,000 replicates are shown along the branches. Scale indicates amino acid substitutions per site.

**Figure 3:**
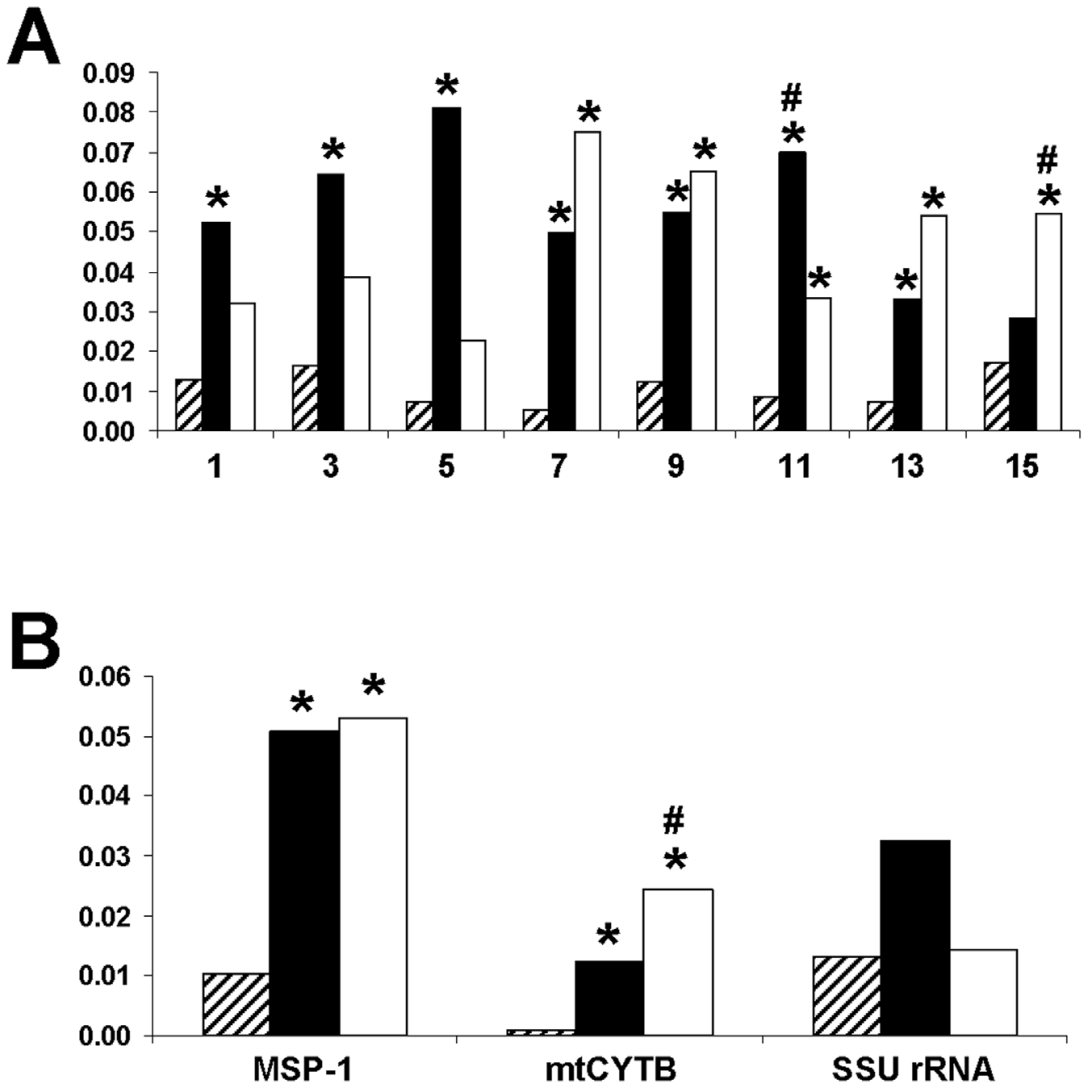
Nucleotide divergence between *P. fieldi* and *P. simiovale* (upward diagonal), *P. ovale curtisi* and *P. ovale wallikeri* (filled) and *P. falciparum* and *P. reichenowi* (unfilled) at each interspecies conserved domain of *MSP-1* (A), entire intersepecies conserved domains of MSP-1, mitochondrial cytochrome b (*mtCYTB*) and small subunit ribosomal RNA (SSU rRNA) (B) based on the number of base substitutions per site between sequences using the maximum composite likelihood model. Asterisks and pound signs indicate significant differences when compared with *P. fieldi/P. simiovale* and between *P. ovale curtisi/P. ovale wallikeri* and *P. falciparum/P. reichenowi*, respectively.

Phylogenetic inference from concatenated amino acid sequences of human, nonhuman primate, murine and avian malarial MSP-1 using the maximum likelihood method has shown that *PoMSP-1* and *MSP-1* of *P. malariae* (*PmMSP-1*) shared a node deep in the tree as previously noted [14]. Importantly, *PocMSP-1* and *PowMSP-1* occupied a distinct bifurcating branch with 100% bootstrap support (Figure 2). It is noteworthy that the branch length from the node of *PocMSP-1* and *PowMSP-1* was longer than that separating *MSP-1* of *P. fieldi* (*PfiMSP-1*) and *P. simiovale* (*PsoMSP-1*). It is of note that the evolutionary divergence based on nucleotide sequences between *PocMSP-1* and *PowMSP-1* was significantly greater than those between *PfiMSP-1* and *PsoMSP-1* in all but one interspecies conserved domain (Figure 3). Likewise, the nucleotide divergences of concatenated interspecies conserved domains between *PfMSP-1* and *MSP-1* of *P. reichenowi* (*PrMSP-1*), and between *PocMSP-1* and *PowMSP-1* were significantly more than that of *PfiMSP-1* and *PsoMSP-1*. Meanwhile, comparison of nucleotide divergence at the *mtCYTB* locus between these closely related malaria species has shown similar findings as those for the *MSP-1* locus. Furthermore, the mean divergence at the *SSU rRNA* locus between *P. ovale curtisi* and *P. ovale wallikeri* exceeded that seen for the other species pair (Figure 3). The average divergence time between *PocMSP-1* and *PowMSP-1* was estimated to be around 4.5±0.07 MYA (95% Highest Posterior Density 0.5 – 7.7 MYA). This would correspond to a rate of 1.6×10^−9^ to 5.4×10^−9^ substitutions per site per year, which is close to previous estimates of the range of nucleotide substitution rates of other malarial genes [23]–[25].

## Discussion

Our recent PCR-based diagnosis of malaria species distribution in Thailand involving 5,044 malaria patients during 2006–2007 and 2008–2009 in major endemic areas have shown that malaria caused by *P. ovale* contributed to 1.03% and 0.13%, respectively, of all *Plasmodium* identified [15], [16]. The low prevalence of *P. ovale* in our studies was not caused by PCR primer-escape detection because our *P. ovale*-specific primers target conserved sequences in the *SSU rRNA* genes of both *P. ovale curtisi* and *P. ovale wallikeri* [15], [16]. However, our recent study has shown that using a more sensitive PCR target such as the mitochondrial cytochrome *b* locus has increased the number of *P. ovale*-positive cases than that using the *SSU rRNA* target because the copy of mt*CYTB* per cell outnumbers that of *SSU rRNA* [26]. This also implies that some *P. ovale* infections, especially those co-infecting with other malaria species, occurring at a very low parasite density could be undiagnosed by *SSU rRNA*-based PCR. Therefore, the actual burden of *P. ovale* infection could be underestimated. Meanwhile, the distribution of *P. ovale* in Thailand exhibited geographic variation with a higher prevalence in endemic areas bordering Myanmar than those bordering Cambodia and Malaysia [15], [16].

Recent molecular analysis of various genetic loci of *P. ovale* from diverse geographic origins has purported that the extant *P. ovale* population contained 2 distinct and non-recombining species designated *P. ovale curtisi* and *P. ovale wallikeri* [4], [7]. Phylogenetic analysis has clearly placed both siblings into distinct bifurcating branch with high bootstrap support. Molecular epidemiological studies have further revealed that both *P. ovale curtisi* and *P. ovale wallikeri* are sympatric in Angola, Congo, Equatorial Guinea, Uganda, Ghana, Bangladesh and Myanmar whereas only *P. ovale curtisi* was previously identified in Thailand [4], [27]–[29]. Herein, analyses of the *mtCYTB* and the *MSP-1* loci have further supported co-existence of *P. ovale curtisi* and *P. ovale wallikeri* in this country. Although the actual prevalence of these siblings species in Thailand could not be determined due to the low prevalence of these parasites, our analysis has identified equal number of these parasites, suggesting that both species has circulated in this country at a comparable frequency.

The MSP-1 sequences of *P. ovale wallikeri* newly identified here were structurally differed from those of *P. ovale curtisi* in terms of number of repeat regions and several nucleotide differences (Figure 1). Like other malarial genes, these repeats could be evolved by slippage-strand mispairing or related mechanisms [30]. However, it is noteworthy that the majority of amino acid differences between *PocMSP-1* and *PowMSP-1* sequences occurred outside interspecies conserved domains of MSP-1. The extent of nucleotide diversity in MSP-1 of *P. ovale curtisi* seems to be greater than that of *P. ovale wallikeri* whereas nucleotide diversity at synonymous sites exceed that of nonsynonymous sites in non-repeat regions of both species although no significant difference was observed. On the other hand, when MSP-1 of both species were considered together, synonymous nucleotide diversity significantly outnumbered nonsynonymous nucleotide diversity, suggesting that purifying selection may shape the pattern of sequence diversity in the MSP-1 locus preceding speciation. Both synonymous and nonsynonymous nucleotide diversity were much greater when both *PocMSP-1* and *PowMSP-1* were included in the computation than when *PocMSP-1* and *PowMSP-1* were considered separately (Table 2). This result is consistent with the hypothesis of Sutherland and Polley [4], [31] that *Poc* and *Pow* entered the human host lineage separately, being sampled from an ancestral *P. ovale* population that was more diverse than either *P. ovale curtisi* or *P. ovale wallikeri* is today.

A phylogenetic tree inferred from the MSP-1 sequences has placed *P. ovale curtisi* and *P. ovale wallikeri* in a distinct bifurcating branch with 100% bootstrap support. It is noteworthy that the branch length from the node separating these sibling species of *P. ovale* seems to be longer than that separating *P. fieldi* and *P. simiovale*. Comparison of sequences in interspecies conserved domains in the MSP-1 locus of some closely related malaria has shown that nucleotide divergence between *P. ovale curtisi* and *P. ovale wallikeri* significantly exceeded that between *P. fieldi* and *P. simiovale* in all but one domain. The overall nucleotide divergence in interspecies conserved domains between *P. ovale curtisi* and *P. ovale wallikeri* was comparable to that between *P. falciparum* and *P. reichenowi*. Therefore, the MSP-1 sequence also supports speciation of these sibling species of *P. ovale* akin to other loci such as *mtCYTB* and *SSU rRNA* [4]. Recent analysis using the *mtCYTB* locus and the gene encoding glyceraldehyde-3-phosphatase has suggested that time to the most recent common ancestor to *P. ovale curtisi* and *P. ovale wallikeri* was between 1.0 and 3.5 MYA [4]. Meanwhile, the divergence time in the *MSP-1* locus of malaria parasites seems to be much more ancient than house-keeping gene loci. The dimorphic prototypes of *PfMSP-1*, represented by K1 and MAD20 strains, seem to share the last common ancestor around 27 - 35 MYA [23], [24] whereas the divergence time between *P. vivax* and *P. knowlesi* coincides with the time of radiation of Southeast Asian macaques about 5 MYA [32], [33]. However, our analysis has suggested that the split of *PocMSP-1*and *PowMSP-1* seems to be relatively more recent. Therefore, the *MSP-1* sequences of *P. ovale curtisi* and *P. ovale wallikeri* support ancient divergence times of malaria lineages [34], [35].

One of the major mechanisms generating genetic diversity in the *MSP-1* locus is interallelic recombination between distinct alleles during malarial sexual development in anopheleine vectors [10], [11]. Epidemiological studies have shown that the degree of heterologous mating in malaria populations is positively correlated with transmission rates and the prevalence of mixed allele infections [36]. In Thailand, the extent of diversity in *PvMSP-1* seems to be highly variable with extensive sequence diversity among isolates from northwestern region where both prevalence and mixed allele infections of *P. vivax* have been more pronounced than other endemic areas of the country [15], [16]. On the other hand, only few *PvMSP-1* alleles were detected in *P. vivax* populations from southern Thailand as a consequence of preceding population bottlenecks, probably from extensive malaria control measures [37]. By contrast, the extent of diversity in both *PocMSP-1* and *PowMSP-1* in this study was at least an order of magnitude lower than that for *PvMSP-1* whereas perfectly identical *PowMSP-1* or *PocMSP-1* sequences were identified in isolates collected over a decade apart. The low level of sequence diversity in both *PocMSP-1* and *PowMSP-1* could stem from repeated genetic bottlenecks, low transmission rate and autogamous breeding. Unfortunately, the limited number of samples in this study precludes a meaningful analysis of genetic recombination at this locus. On the other hand, the prevalence of *P. ovale* in Thailand has been persistently low despite using molecular detection [15], [16]; thereby, limiting the chance for heterozygous mating comparing with other malaria species.

In conclusion, the nucleotide divergence of the *MSP-1* sequences support that *P. ovale curtisi* and *P. ovale wallikeri* are distinct species. The low level of within species diversity at the *MSP-1* sequences could be a result of low transmission rate and repeated bottleneck effects.
